# Supplementary material for: MeCP2 binds to methylated DNA independently of phase separation and heterochromatin organisation
Source: Nat Commun. 2024 May 8;15:3880. doi: 10.1038/s41467-024-47395-1 (PMC11079052; doi:10.1038/s41467-024-47395-1)

Supplementary Information

MeCP2 binds to methylated DNA independently of phase separation and heterochromatin organisation

Raphaël Pantier[1], Megan Brown[1], Sicheng Han[1], Katie Paton[1], Stephen Meek[2], Thomas Montavon[3], Nicholas Shukeir[3], Toni McHugh[1], David A. Kelly[1], Tino Hochepied[4,5], Claude Libert[4,5], Thomas Jenuwein[3], Tom Burdon[2], Adrian Bird[1*]

[1] The Wellcome Centre for Cell Biology, University of Edinburgh, Michael Swann Building, Max Born Crescent, The King's Buildings, Edinburgh, EH9 3BF, UK

[2] The Roslin Institute and Royal (Dick) School of Veterinary Studies, University of Edinburgh, Easter Bush, Midlothian, EH25 9RG, UK

[3] Max Planck Institute of Immunobiology and Epigenetics, Stübeweg 51, 79108 Freiburg, Germany

[4] Center for Inflammation Research, VIB, Ghent, Belgium

[5] Department of Biomedical Molecular Biology, Ghent University, Ghent, Belgium

* Corresponding author: a.bird@ed.ac.uk

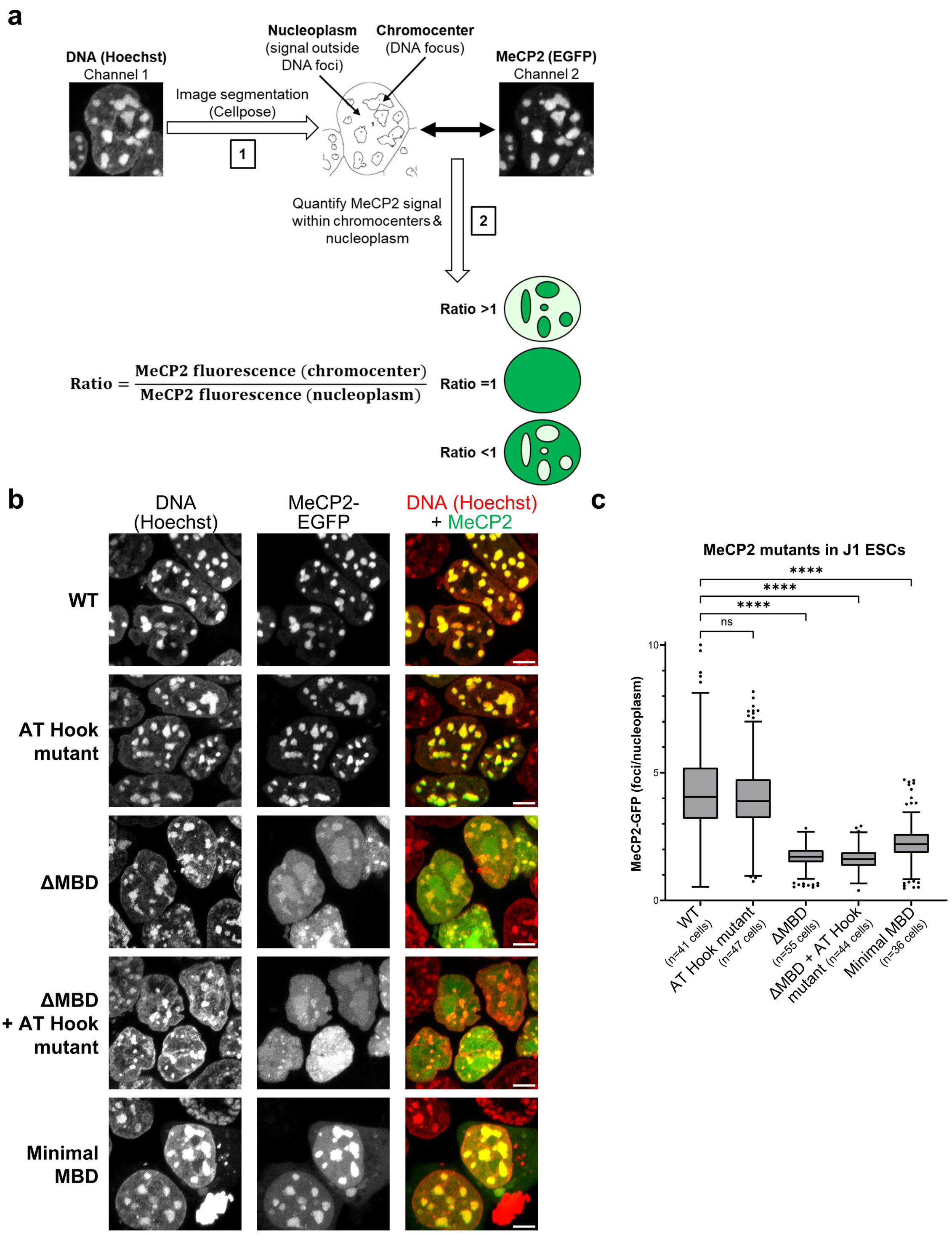

Supplementary Fig. 1 (related to Fig. 1) | a. Diagram showing the strategy for quantifying the relative enrichment of MeCP2 within chromocenters (DNA-dense foci) in mouse cells. In each cell, the fluorescence ratio was calculated for each individual chromocenter. **b.** Live-cell imaging of J1 ESCs transfected with EGFP-MeCP2 wild-type and mutant constructs (see Fig. 1a). Hoechst staining was used to visualise DNA. Scale bars: 5μm. **c.** Box plot showing the quantification of wild-type and mutant MeCP2 fluorescence at DNA-dense foci (relative to nucleoplasm) in J1 ESCs, as described in panel b. The box lower and upper limits correspond to the 25th and 75th percentiles, respectively, with the centre line corresponding to the median. Whiskers extend up to 1.5 times the inter-quartile distance according to Tukey's method, and individual points are outliers. The number of analysed cells from two independent experiments are: WT n= 41 cells, AT Hook mutant n= 47 cells, ΔMBD n= 55 cells, ΔMBD + AT Hook mutant n= 44 cells, Minimal MBD n= 36 cells. Stars indicate statistical significance compared to wild-type MeCP2 (Brown-Forsythe and Welch ANOVA test). The Source data for this Supplementary Figure is associated with the Source data for Figure 1.

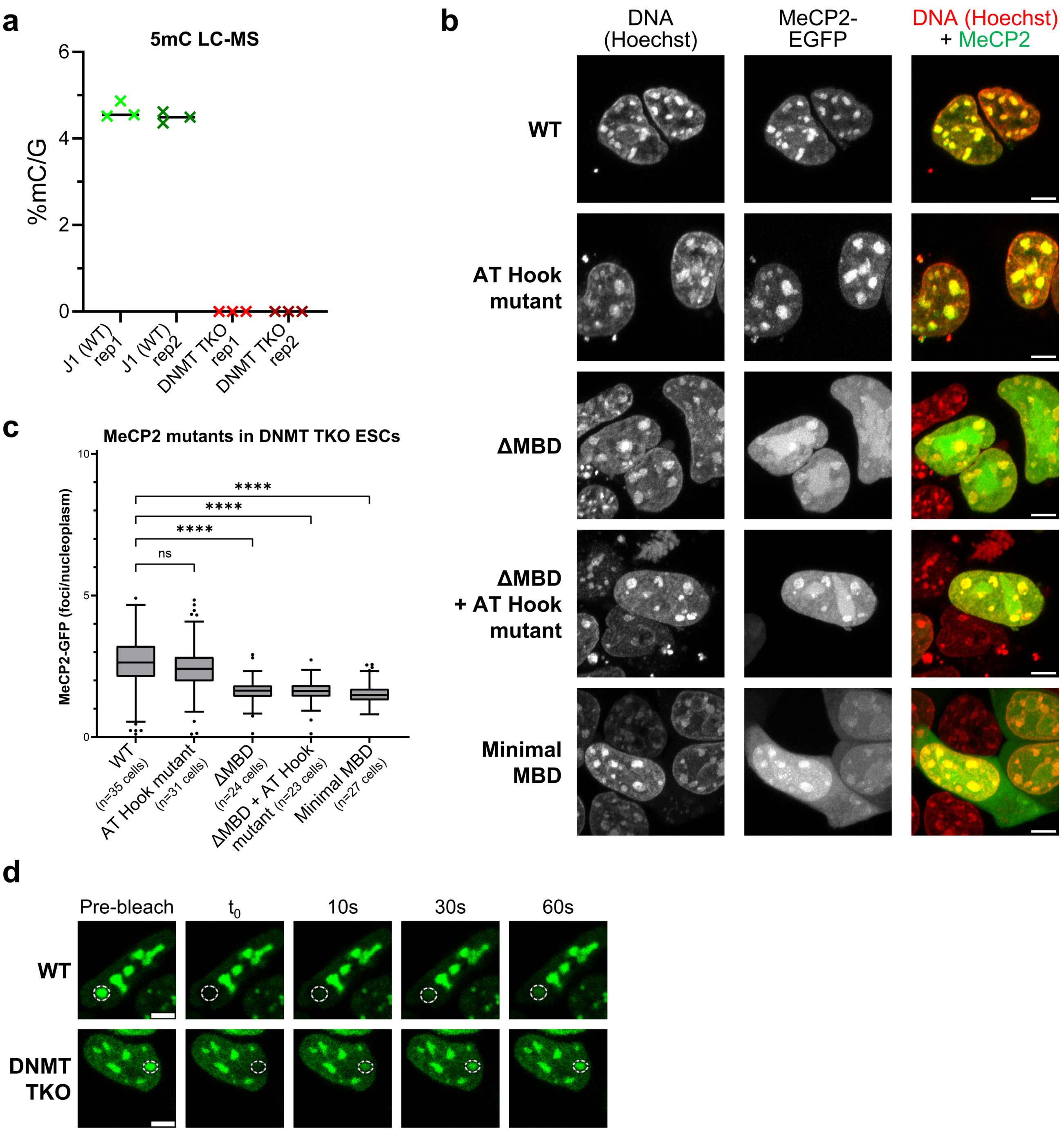

Supplementary Fig. 2 (related to Fig. 1) | a. Mass spectrometric quantification of 5-methylcytosine (relative to total guanosine pool) from genomic DNA in the indicated ESC lines (two independent replicates). Individual data points are technical replicates and lines correspond to the median. **b.** Live-cell imaging of *DNMT TKO* ESCs transfected with EGFP-MeCP2 wild-type and mutant constructs (see Fig. 1a). Hoechst staining was used to visualise DNA. Scale bars: 5µm. **c.** Box plot showing the quantification of wild-type and mutant MeCP2 fluorescence at DNA-dense foci (relative to nucleoplasm) in *DNMT TKO* ESCs, as described in panel b. The box lower and upper limits correspond to the 25th and 75th percentiles, respectively, with the centre line corresponding to the median. Whiskers extend up to 1.5 times the inter-quartile distance according to Tukey's method, and individual points are outliers. The number of analysed cells from two independent experiments are: WT n= 35 cells, AT Hook mutant n= 31 cells, ΔMBD n= 24 cells, ΔMBD + AT Hook mutant n= 23 cells, Minimal MBD n= 27 cells. Stars indicate statistical significance compared to wild-type MeCP2 (Brown-Forsythe and Welch ANOVA test). **d.** Live-cell imaging showing the fluorescence recovery after photobleaching (FRAP) of wild-type EGFP-MeCP2 in wild-type (J1) and *DNMT TKO* ESCs. Scale bars: 5µm. The Source data for this Supplementary Figure is associated with the Source data for Figure 1.

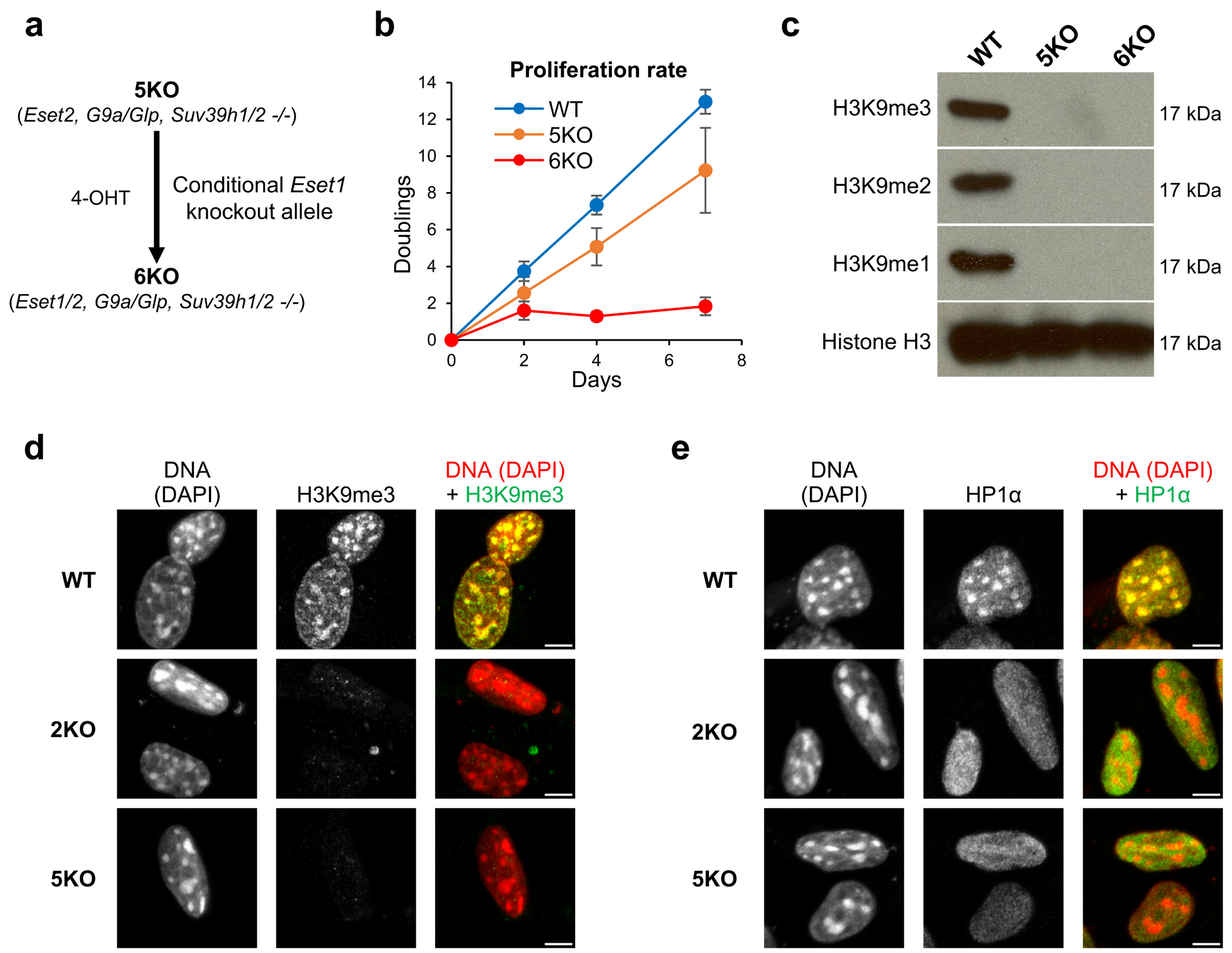

Supplementary Fig. 3 (related to Fig. 2) | a. Diagram showing the genotype of H3K9 lysine methyltransferase knockout cell lines. *5KO* fibroblasts lack five out of six methyltransferase genes and can be converted to a *6KO* state upon tamoxifen induction. **b.** Growth curves showing that Eset1 knockout (*6KO*) leads to a lethal phenotype within 2 to 4 days. Error bars: SD. **c.** Western blot analysis of canonical heterochromatin marks H3K9me1/2/3 in the indicated cell lines. Total histone H3 was probed as a positive control. **d, e.** Immunofluorescence of H3K9me3 (d) and HP1α (e) in wild-type (Eset25) and H3K9 lysine methyltransferases knockout fibroblasts (*2KO*/*5KO*). DAPI staining was used to visualise DNA. Scale bars: 5μm. The Source data for this Supplementary Figure is associated with the Source data for Figure 2.

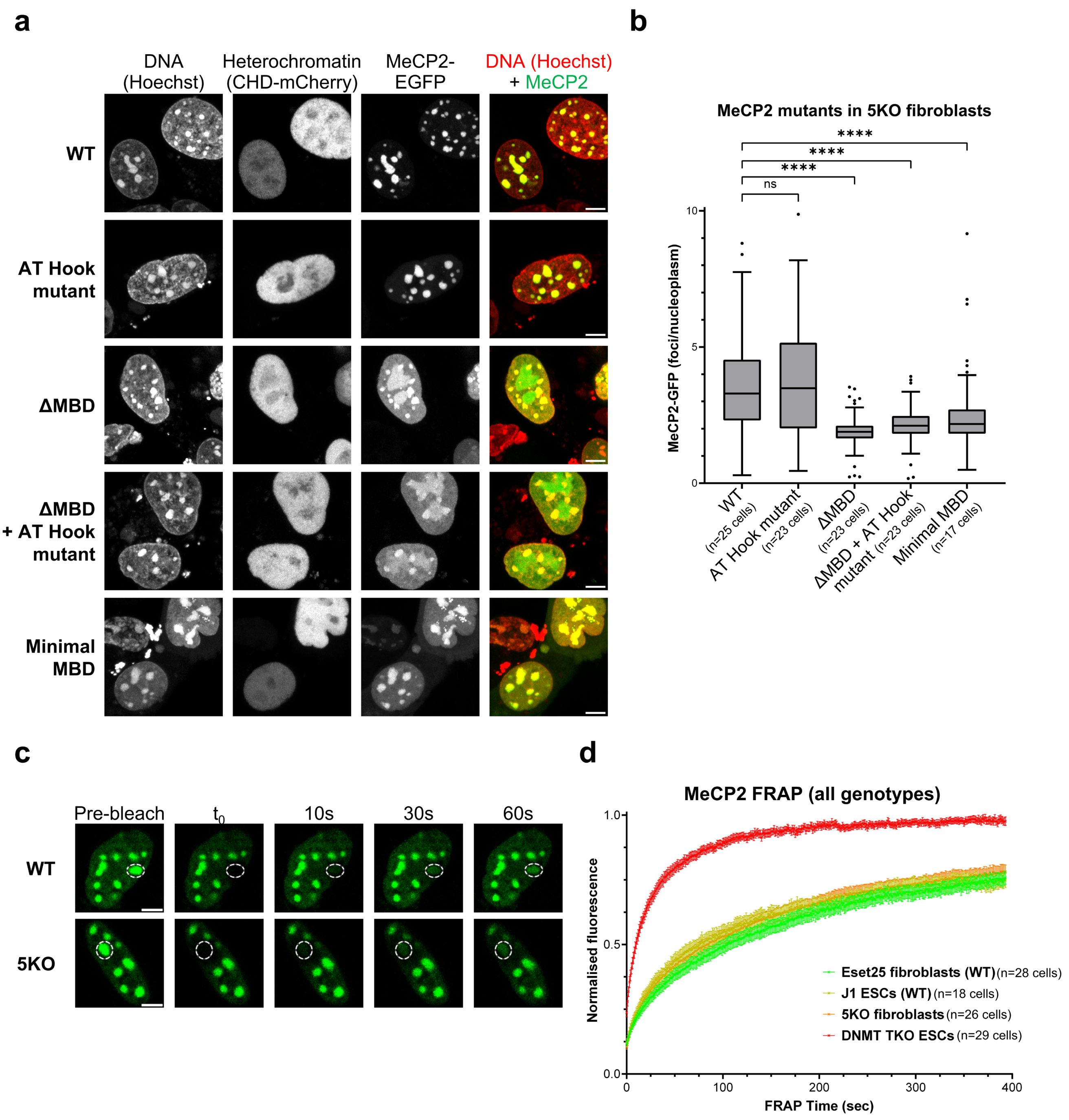

Supplementary Fig. 4 (related to Fig. 2) | a. Live-cell imaging of *5KO* fibroblasts transfected with EGFP-MeCP2 wild-type and mutant constructs (see Fig. 1a). Hoechst staining and CHD-mCherry reporter were used to visualise DNA and heterochromatin, respectively. Scale bars: 5μm. **b.** Box plot showing the quantification of MeCP2 wild-type and mutant fluorescence at DNA-dense foci (relative to nucleoplasm) in *5KO* fibroblasts, as described in panel a. The box lower and upper limits correspond to the 25th and 75th percentiles, respectively, with the centre line corresponding to the median. Whiskers extend up to 1.5 times the inter-quartile distance according to Tukey's method, and individual points are outliers. The number of analysed cells from two independent experiments are: WT n= 25 cells, AT Hook mutant n= 23 cells, ΔMBD n= 23 cells, ΔMBD + AT Hook mutant n= 23 cells, Minimal MBD n= 17 cells. Stars indicate statistical significance compared to wild-type MeCP2 (Brown-Forsythe and Welch ANOVA test). **c.** Live-cell imaging showing the fluorescence recovery after photobleaching (FRAP) of wild-type EGFP-MeCP2 in wild-type (Eset25) and *5KO* fibroblasts. Scale bars: 5μm. **d.** Graph showing the FRAP quantification of wild-type EGFP-MeCP2 in all mutant lines used in this study (see Fig. 1 and 2). The number of analysed cells from two independent experiments are: Eset25 (WT) n= 28 cells, J1 (WT) n= 18 cells, *5KO* n= 26 cells, *DNMT TKO* n= 29 cells. Error bars: SEM. The Source data for this Supplementary Figure is associated with the Source data for Figure 2.

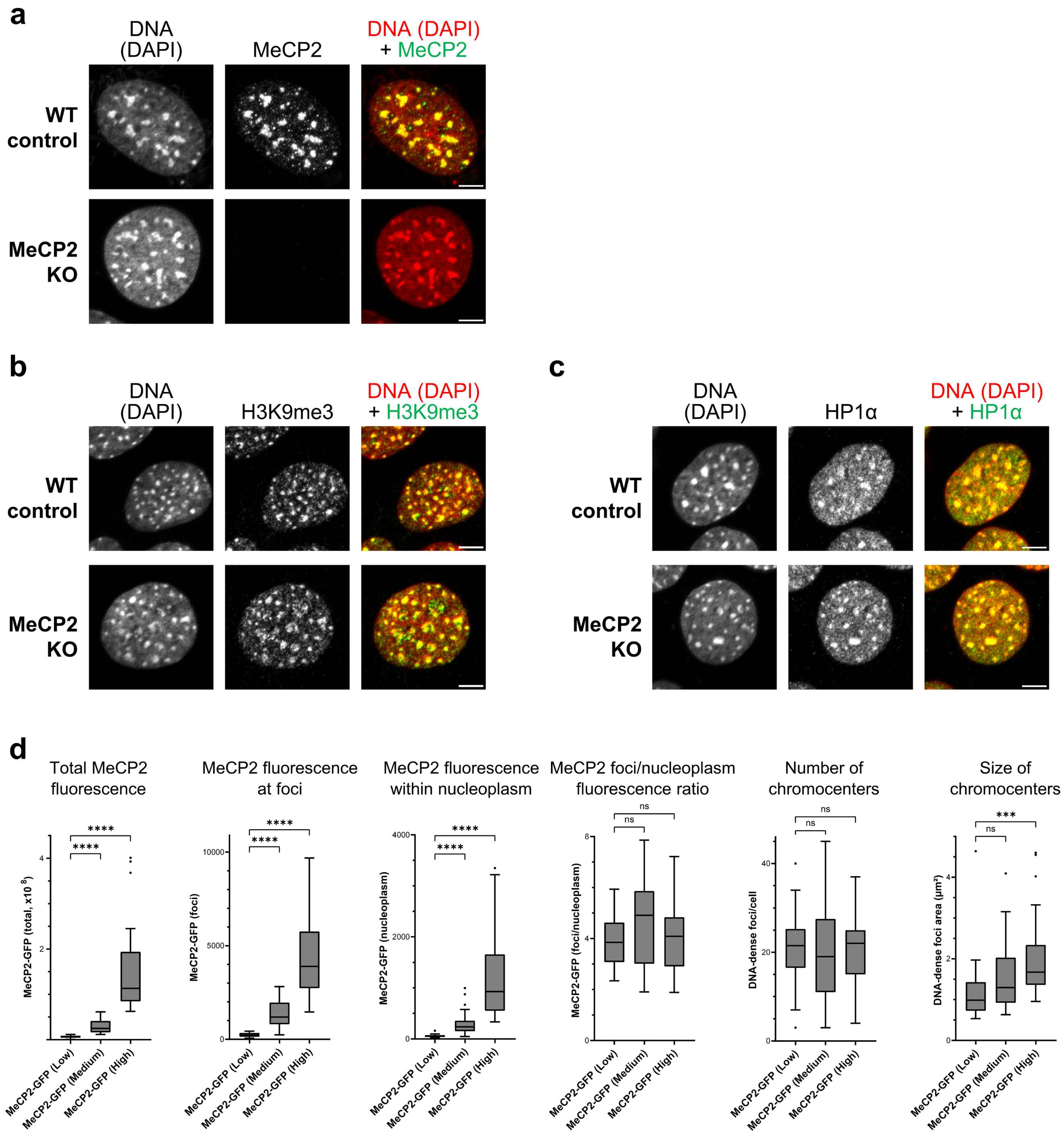

Supplementary Fig. 5 (related to Fig. 2) | a, b, c. Immunofluorescence of MeCP2 (a), H3K9me3 (b) and HP1α (c) in wild-type (2-17) and *MeCP2* knockout fibroblasts. DAPI staining was used to visualise DNA. Scale bars: 5µm. **d.** Box plots showing the quantification of different parameters in transfected *Mecp2* knockout fibroblasts (see Fig. 2f) divided into three categories depending on their MeCP2 expression levels (low: n=34 cells; medium: n=33 cells; high: n=34 cells). The box lower and upper limits correspond to the 25th and 75th percentiles, respectively, with the centre line corresponding to the median. Whiskers extend up to 1.5 times the inter-quartile distance according to Tukey's method, and individual points are outliers. Stars indicate statistical significance between groups of MeCP2 expression level (Brown-Forsythe and Welch ANOVA test). The Source data for this Supplementary Figure is associated with the Source data for Figure 2.

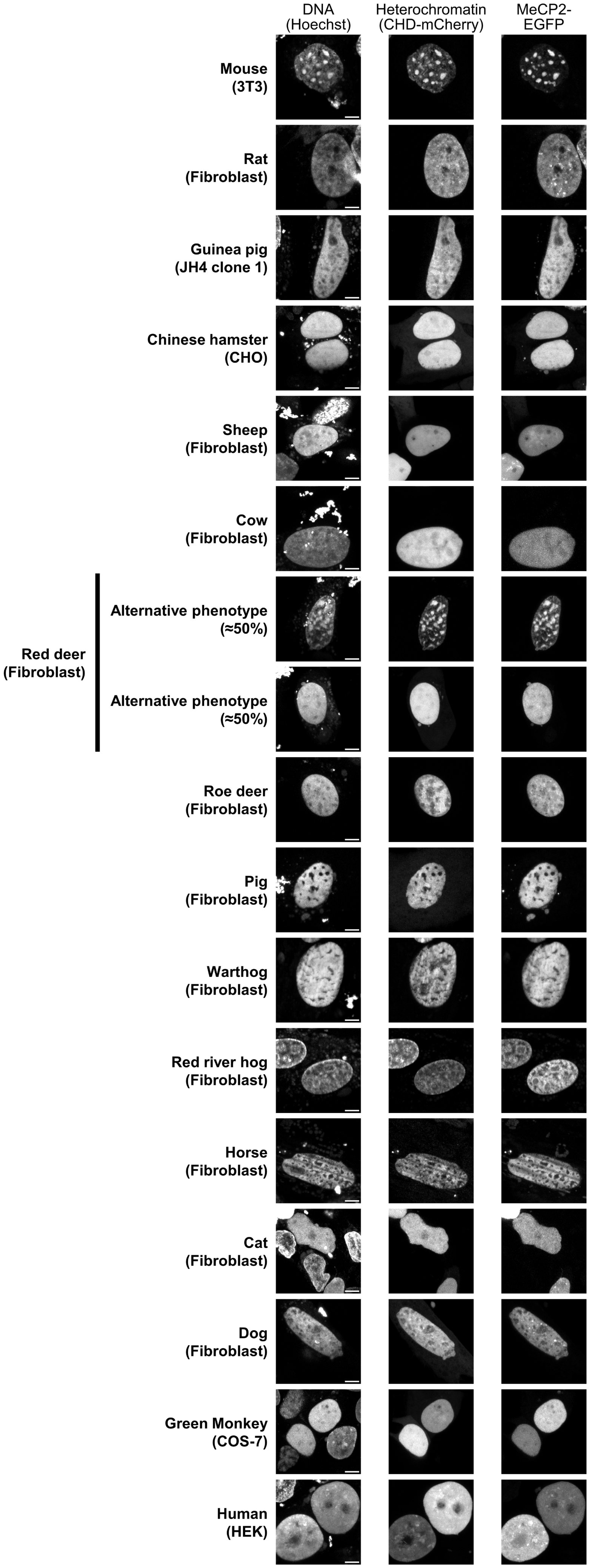
DNA (Hoechst)
Heterochromatin (CHD-mCherry)
MeCP2-EGFP
Mouse (3T3)
Rat (Fibroblast)
Guinea pig (JH4 clone 1)
Chinese hamster (CHO)
Sheep (Fibroblast)
Cow (Fibroblast)
Red deer (Fibroblast)
Alternative phenotype (≈50%)
Alternative phenotype (≈50%)
Roe deer (Fibroblast)
Pig (Fibroblast)
Warthog (Fibroblast)
Red river hog (Fibroblast)
Horse (Fibroblast)
Cat (Fibroblast)
Dog (Fibroblast)
Green Monkey (COS-7)
Human (HEK)

Supplementary Fig. 6 (related to Fig. 3) | Live-cell imaging of all studied mammalian cell lines transfected with wild-type EGFP-MeCP2. Hoechst staining and CHD-mCherry reporter were used to visualise DNA and heterochromatin, respectively. Scale bars: 5µm. Of note, red deer cells presented a mixed population with two distinct phenotypes characterised by diffuse or spotty MeCP2/Heterochromatin, respectively. The Source data for this Supplementary Figure is associated with the Source data for Figure 3.

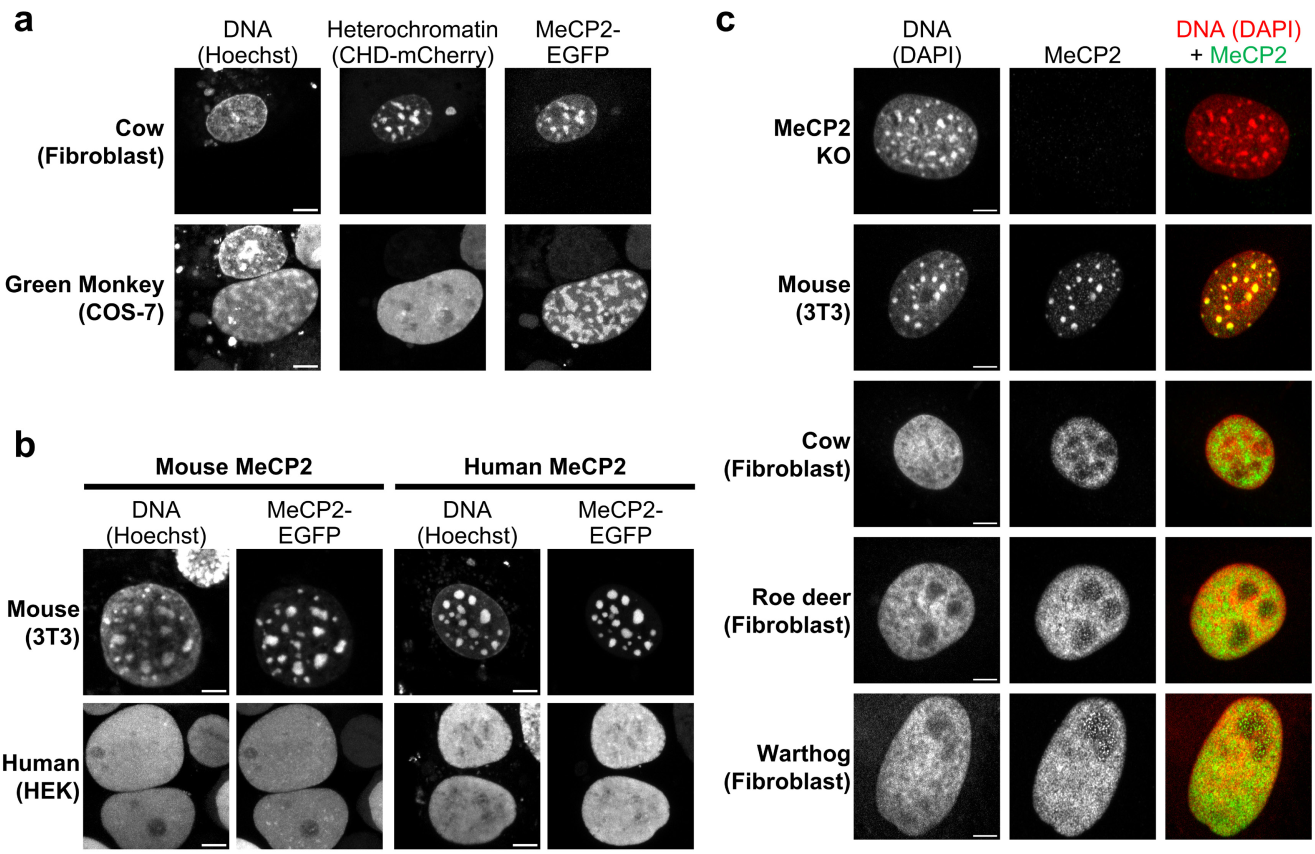

Supplementary Fig. 7 (related to Fig. 3) | a. Live-cell imaging showing a rare sub-population of cow and monkey cells with spotty signal when transfected with wild-type EGFP-MeCP2 (see Fig. 3c). Hoechst staining and CHD-mCherry reporter were used to visualise DNA and heterochromatin, respectively. Scale bars: 5µm. **b.** Live-cell imaging of mouse (3T3) and human (HEK) cell lines transfected with mouse (left) or human (right) wild-type EGFP-MeCP2. Hoechst staining was used to visualise DNA. Scale bars: 5µm. **c.** Immunofluorescence of endogenous MeCP2 in the indicated mammalian cell lines and *MeCP2* knockout fibroblasts (negative control). DAPI staining was used to visualise DNA. Scale bars: 5µm. The Source data for this Supplementary Figure is associated with the Source data for Figure 3.

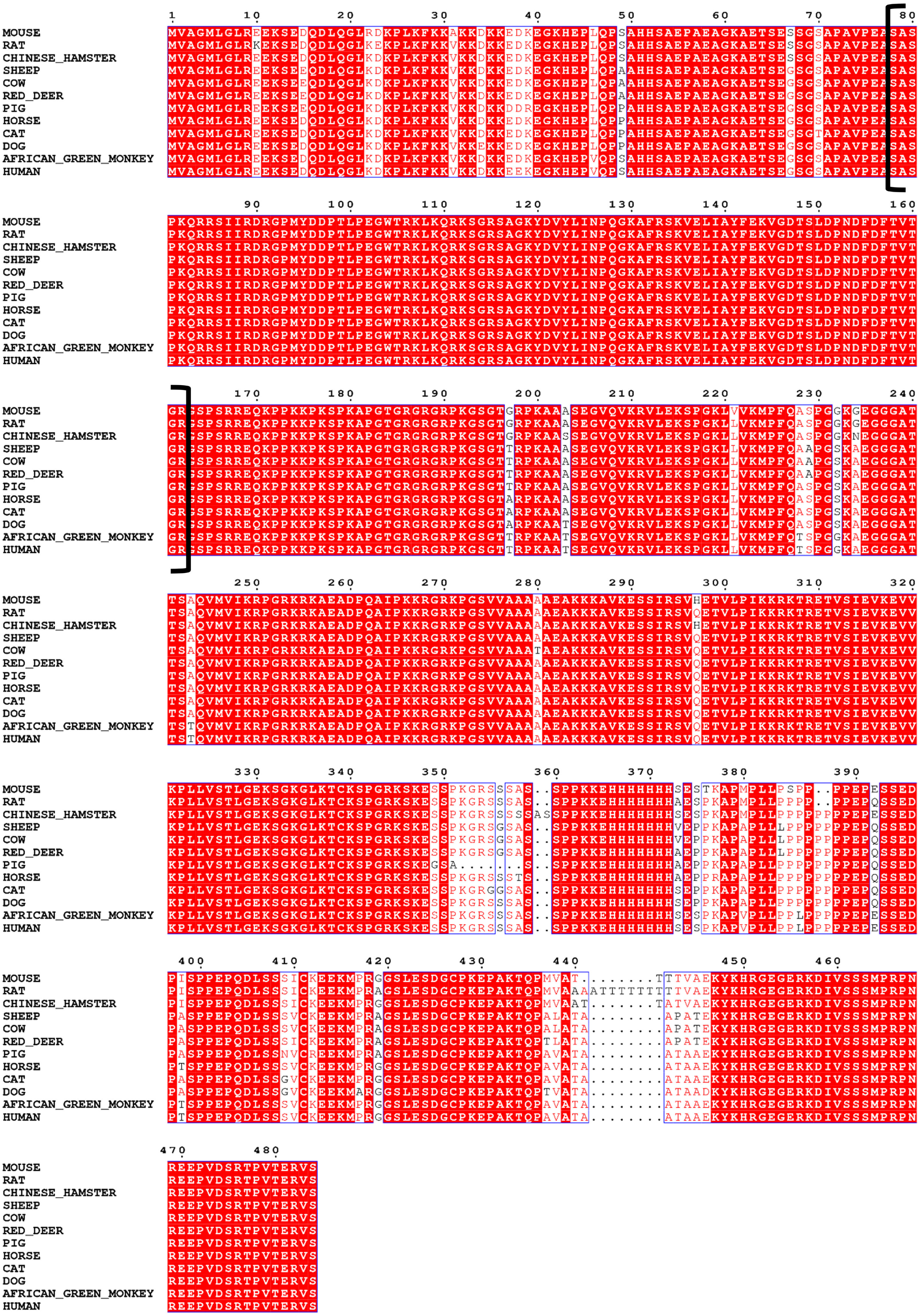

Supplementary Fig. 8 (related to Fig. 3) | Protein alignment of MeCP2 across mammalian species. Identical residues are white on a red background; conservative substitutions found in several mammalian species are in red text with white background. Brackets indicate the MBD domain (residues 78-162) which is strictly conserved. The Source data for this Supplementary Figure is associated with the Source data for Figure 3.

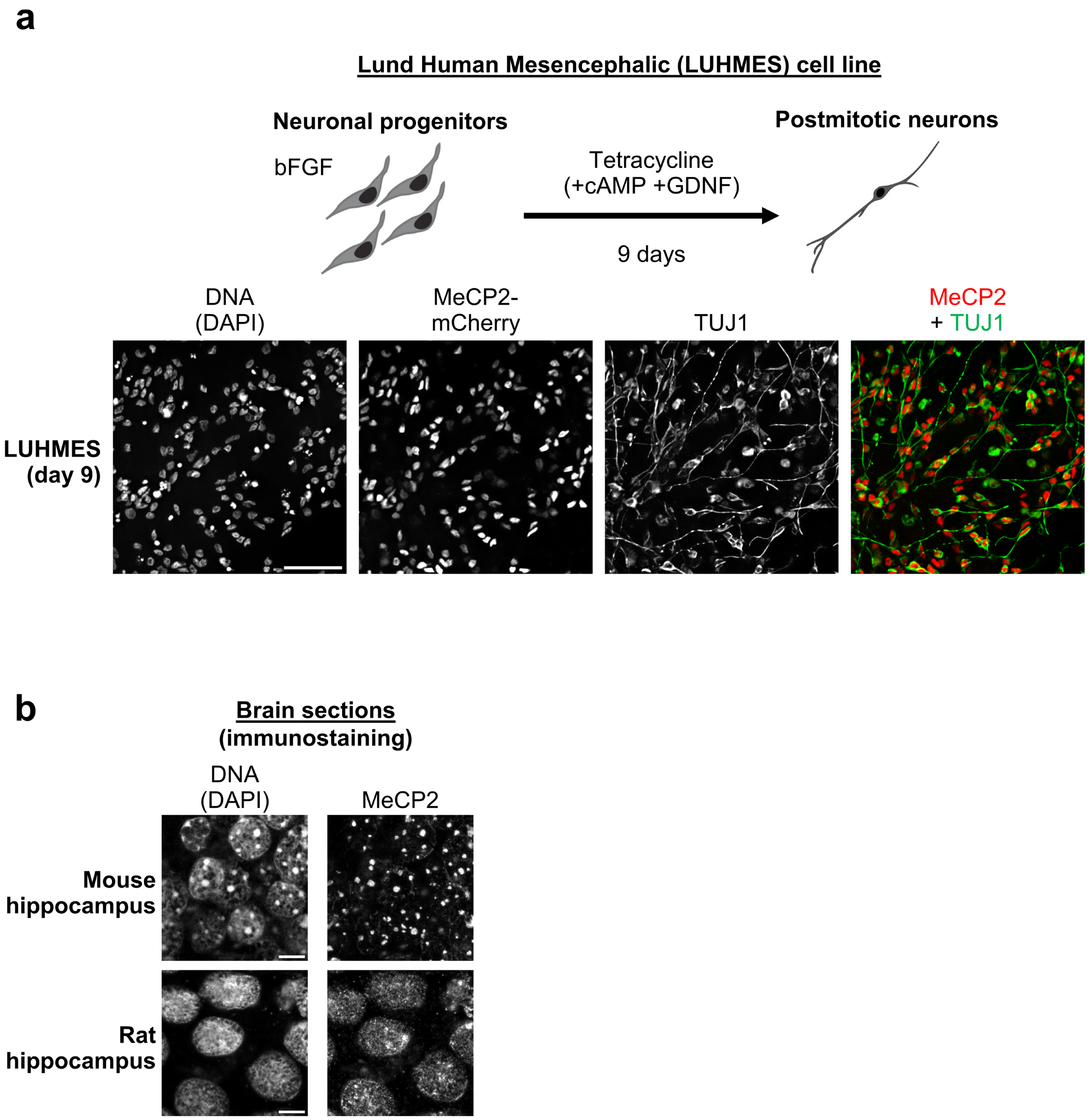

Supplementary Fig. 9 (related to Fig. 3) | a. Immunofluorescence of TUJ1 in human postmitotic neurons (LUHMES) expressing endogenously tagged MeCP2-mCherry. DAPI staining was used to visualise DNA. Scale bar: 50µm. Diagram adapted from Shah et al, Wellcome Open Res, 2016 under the terms of a Creative Commons Attribution License (https://creativecommons.org/licenses/by/4.0). **b.** Immunofluorescence of endogenous MeCP2 in mouse and rat brain sections (hippocampus). DAPI staining was used to visualise DNA. Scale bars: 5µm. The Source data for this Supplementary Figure is associated with the Source data for Figure 3.

a

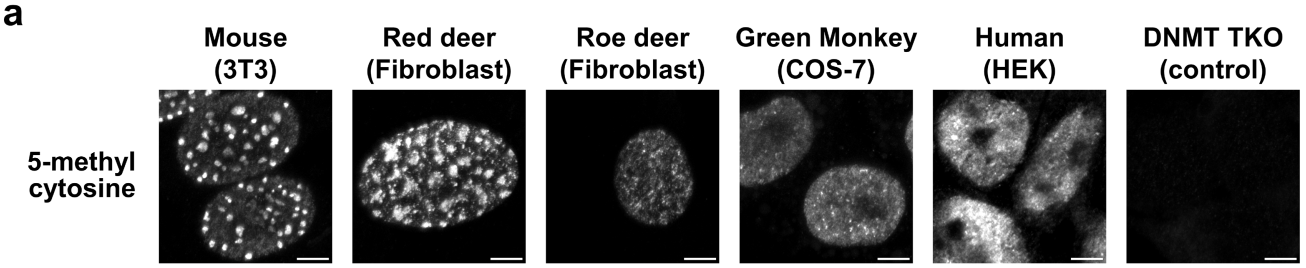

b

```
5' CAAGACGAAAGGATGTCTGAATCCCCTGTGGAGACCACAGAGAAAGACCTAGTTCCCCACCTCATC
3' GTTCTGCTTTCCTACAGACTTAGGGGACACCTCTGGTGTCTCTTTCTGGATCAAGGGGTGGAGTAG
       HpaII/MspI
   GCGACCGGAGGCCTCACATCCTTTGAAAACTCCAGAGGTACGCGGAGATCAGTGCCTCCAAAGGAG
   CGCTGGCCTCCGGAGTGTAGGAAACTTTTGAGGTCTCCATGCGCCTCTAGTCACGGAGGTTTCCTC

   ACGATGCCTGACTCCTCGTGAAACTTGATAGGAGTCCCAGGATTCCTGTGGCACGTGGAAAGGGAC
   TGCTACGGACTGAGGAGCACTTTGAACTATCCTCAGGGTCCTAAGGACACCGTGCACCTTTCCCTG

   CCTTGGTCTCCCGCCTCAGCTGGAGAGGCGTCCCAATTGCCCTGCCAAGCCTCGAGGAGAATCCCG
   GGAACCAGAGGGCGGAGTCGACCTCTCCGCAGGGTTAACGGGACGGTTCGGAGCTCCTCTTAGGGC

   AGTTGTCCCTCGCAACTAGGCAGGAGTCCTGACGTCGCTGAAGAAACACGTGTGTGGAAGGGCCAT
   TCAACAGGGAGCGTTGATCCGTCCTCAGGACTGCAGCGACTTCTTTGTGCACACACCTTCCCGGTA

   CCCCGTCGTAACTCGAGAATATACCCCAGGTTCCCGCCGCAACTCGAGAAAAACCATGAGACTTCC
   GGGGCAGCATTGAGCTCTTATATGGGGTCCAAGGGCGGCGTTGAGCTCTTTTTGGTACTCTGAAGG

   CCCTCGCCGCGAGATGAGGCCCGATTCCCCTGCACTGCGTGCAGAGCAATTCCGTGTTGCACATCA
   GGGAGCGGCGCTCTACTCCGGGCTAAGGGGACGTGACGCACGTCTCGTTAAGGCACAACGTGTAGT

   CACATGAAAGGAGCCTTGATTTCCTTGATGGCACTCCAGAGAAACCCCAAGAACACTGTTTCAAGG
   GTGTACTTTCCTCGGAACTAAAGGAACTACCGTGAGGTCTCTTTGGGGTTCTTGTGACAAAGTTCC

   CTAGAGGGATCCTGAGGTCACTGTAGCAACACGAAAGAGCTCCGTGGACCAAAAATCAACTCGAGA
   GATCTCCCTAGGACTCCAGTGACATCGTTGTGCTTTCTCGAGGCACCTGGTTTTTAGTTGAGCTCT

   TGAGAGGTTAGTCCCTGGCTTCGACTCCAGAGGAATACCACCTTACCACAAGCACCTCAAGAGGAG
   ACTCTCCAATCAGGGACCGAAGCTGAGGTCTCCTTATGGTGGAATGGTGTTCGTGGAGTTCTCCTC

   GCTTCTCTCAGCTCTAGGTATGTGAGAGGGACCCTGAGTTTGCGGCCTCAAGTGGAATGGACACC 3'
   CGAAGAGAGTCGAGATCCATACACTCTCCCTGGGACTCAAACGCCGGAGTTCACCTTACCTGTGG 5'
```

c

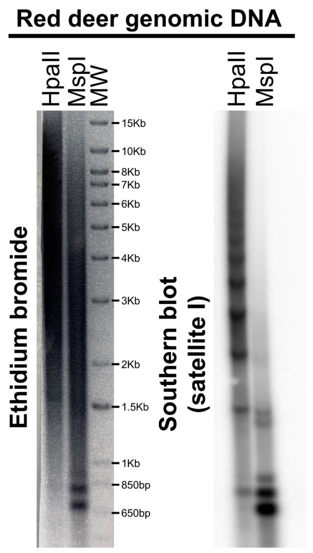

d

```
                                                              HpyCH4IV
5' GGACCTGGAATATGGCGAGAAAACTGAAAATCACGGAAAATGAGAAATACACACTTTAGGACGT
3' CCTGGACCTTATACCGCTCTTTTGACTTTTAGTGCCTTTTACTCTTTATGTGTGAAATCCTGCA
                                        ApoI               HpyCH4IV
   GAAATATGGCGAGGAAAACTGAAAAAGGTGGAAAATTTAGAAATGTCCACTGTAGGACGTGGAA
   CTTTATACCGCTCCTTTTGACTTTTTCCACCTTTTAAATCTTTACAGGTGACATCCTGCACCTT

   TATGGCAAGAAAACTGAAAATCATGGAAAATGAGAAACATCCACTTGACGACTTGAAAAATGAC
   ATACCGTTCTTTTGACTTTTAGTACCTTTTACTCTTTGTAGGTGAACTGCTGAACTTTTTACTG
              HpyCH4IV
   GAAATCACTAAAAAACGTGAAAAATGAGAAATGCACACTGAA 3'
   CTTTAGTGATTTTTTGCACTTTTTACTCTTTACGTGTGACTT 5'
```

Supplementary Fig. 10 (related to Fig. 4) | a. Immunofluorescence of 5-methylcytosine in the indicated mammalian cell lines. Scale bars: 5µm. **b.** Consensus sequence of red deer satellite I. The restriction site for HpaII/MspI enzymes used for Southern blot is highlighted in orange. **c.** Ethidium bromide staining (left) and Southern blot (right) using a probe for satellite I DNA repeats with red deer genomic DNA digested with a methylation-sensitive (HpaII) or -insensitive (MspI) restriction enzyme. Discrete bands at the bottom of the gel with MspI, but not HpaII, digested DNA indicates abundant and highly methylated DNA repeats in red deer cells. MW: Molecular weight marker. **d.** Consensus sequence of mouse major satellite DNA. The restriction sites for HpyCH4IV and ApoI enzymes used for Southern blot are highlighted in red and blue, respectively. The Source data for this Supplementary Figure is associated with the Source data for Figure 4.

Figure 4C

Left panel
(Gel, ethidium bromide staining)

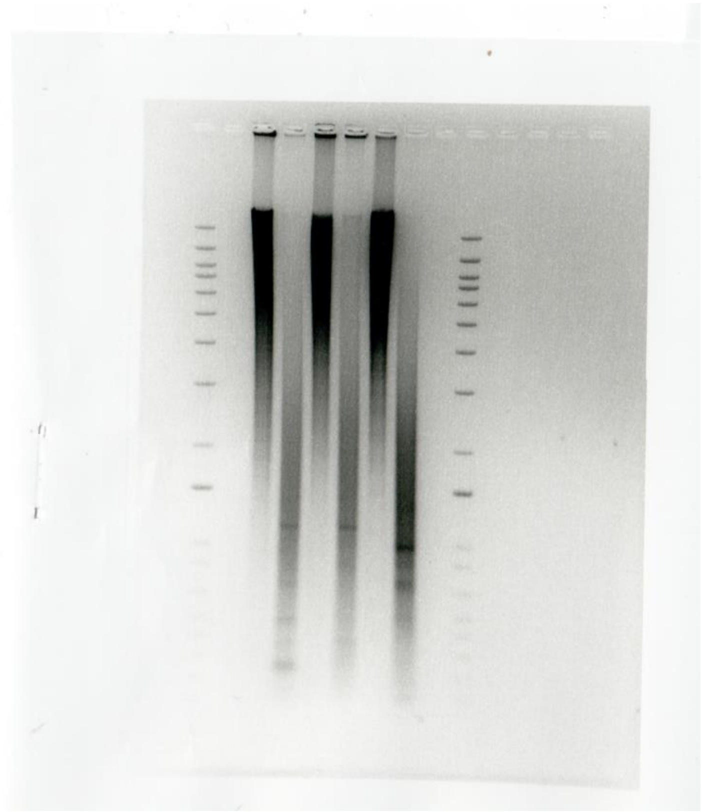

Right panel
(Southern blot, satellite DNA probe)

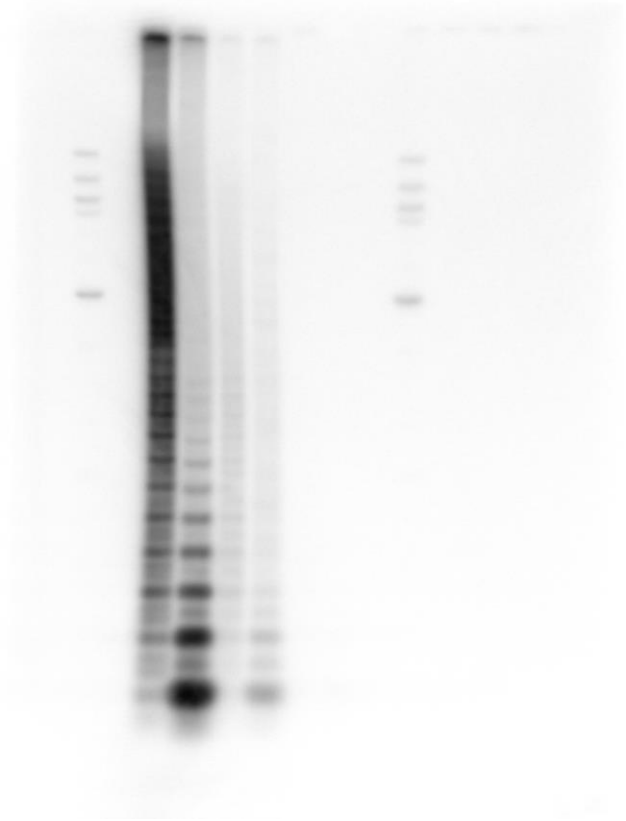

Supplementary Figure 3C (related to Figure 2)

Western blot

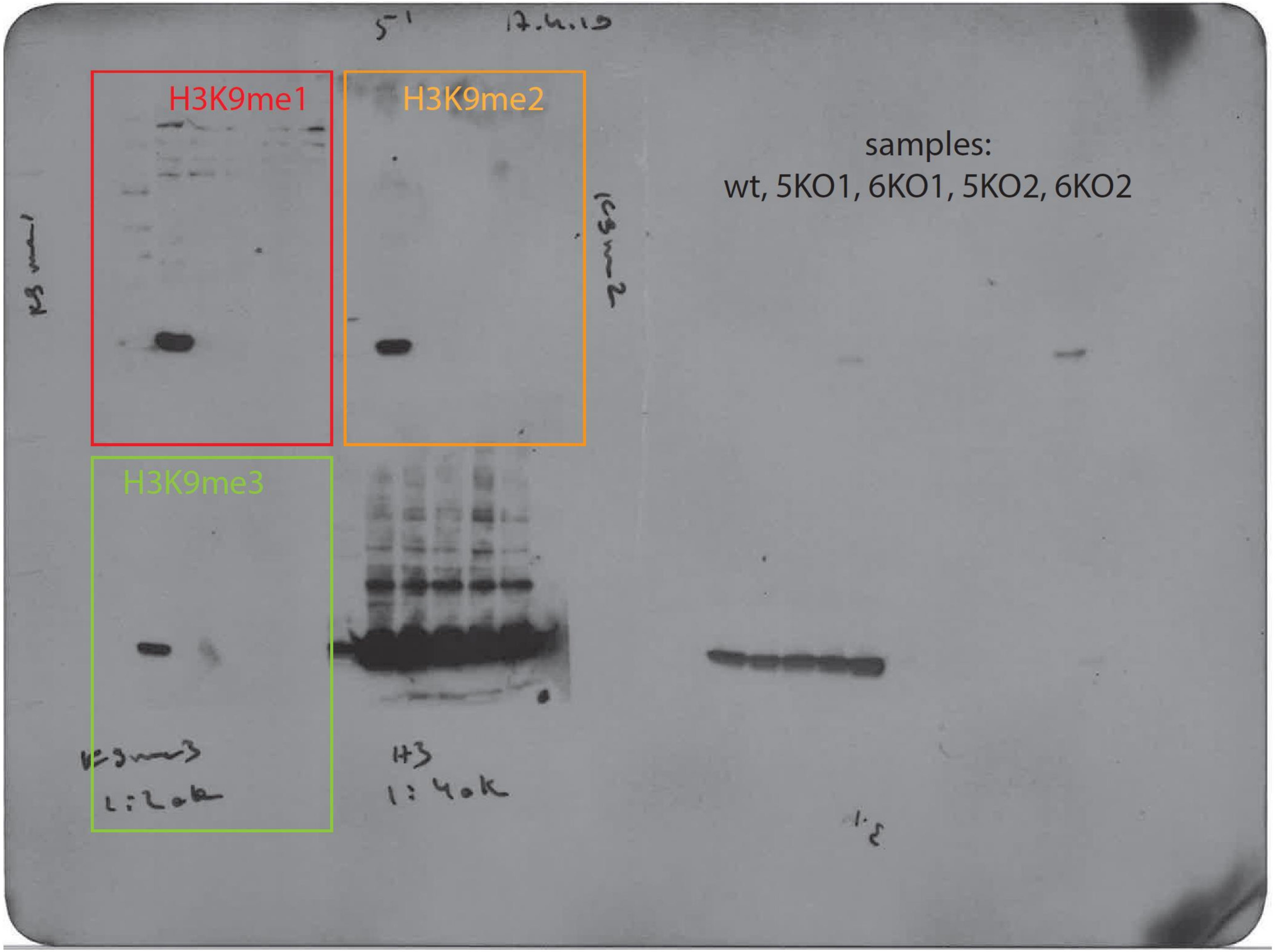

Supplementary Figure 3C
(related to Figure 2)

Western blot

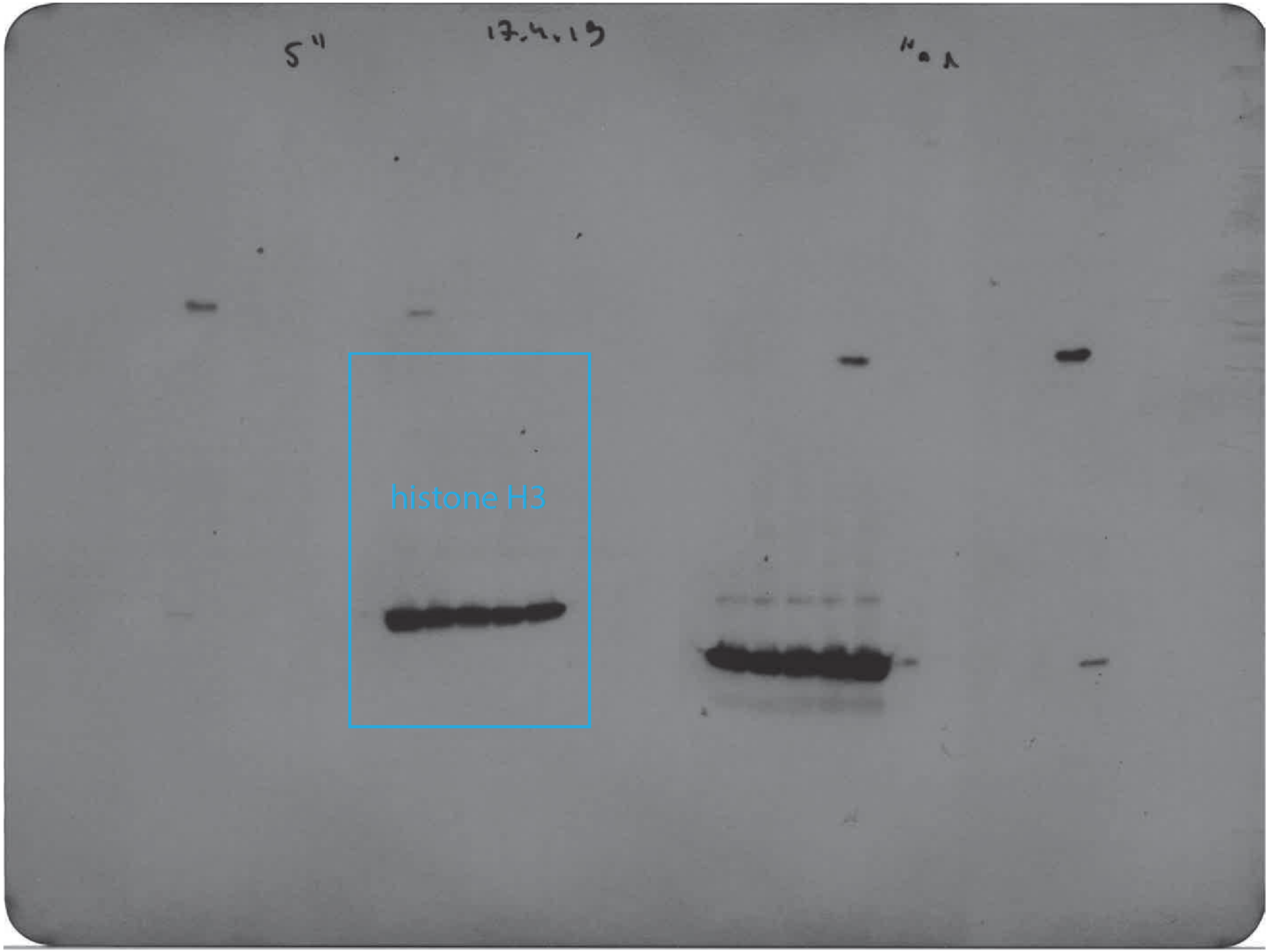

Supplementary Figure 10C
(related to Figure 4)

Left panel
(Gel, ethidium bromide staining)

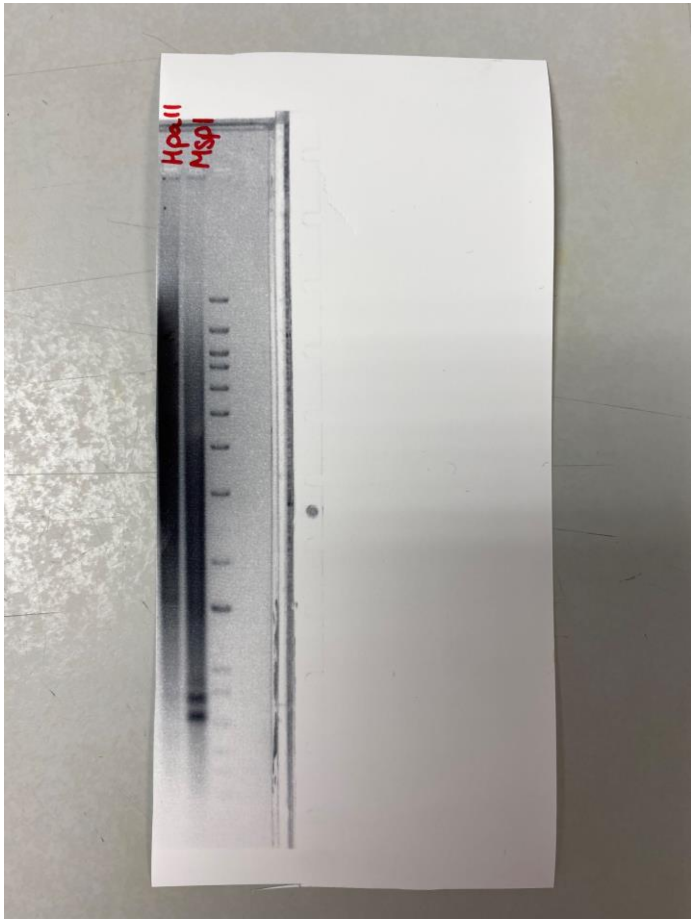

Right panel
(Southern blot, satellite DNA probe)

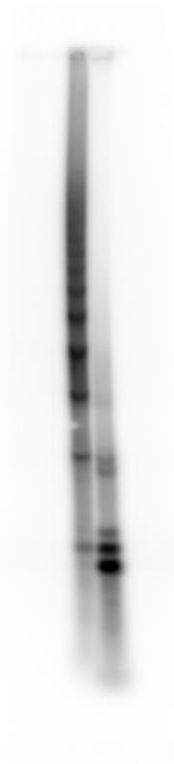

Supplement: Supplementary file 1 — Supplementary Information [file 41467_2024_47395_MOESM1_ESM.pdf]
