# Supplementary material for: MeCP2 binds to methylated DNA independently of phase separation and heterochromatin organisation
Source: Nat Commun. 2024 May 8;15:3880. doi: 10.1038/s41467-024-47395-1 (PMC11079052; doi:10.1038/s41467-024-47395-1)
Supplement: Supplementary file 3 — Description of Additional Supplementary Files [file 41467_2024_47395_MOESM3_ESM.pdf]

Description of Additional Supplementary Files

File Name: Supplementary Data 1
Description: List of cell lines used in the study
