# Supplementary material for: MeCP2 binds to methylated DNA independently of phase separation and heterochromatin organisation
Source: Nat Commun. 2024 May 8;15:3880. doi: 10.1038/s41467-024-47395-1 (PMC11079052; doi:10.1038/s41467-024-47395-1)
Supplement: Supplementary file 5 — Reporting Summary [file 41467_2024_47395_MOESM5_ESM.pdf]

nature portfolio

Corresponding author(s): Adrian Bird

Last updated by author(s): Mar 7, 2024

Reporting Summary

Nature Portfolio wishes to improve the reproducibility of the work that we publish. This form provides structure for consistency and transparency in reporting. For further information on Nature Portfolio policies, see our Editorial Policies and the Editorial Policy Checklist.

Statistics

For all statistical analyses, confirm that the following items are present in the figure legend, table legend, main text, or Methods section.

n/a	Confirmed	
☐	☒	The exact sample size (*n*) for each experimental group/condition, given as a discrete number and unit of measurement
☐	☒	A statement on whether measurements were taken from distinct samples or whether the same sample was measured repeatedly
☐	☒	The statistical test(s) used AND whether they are one- or two-sided<br>*Only common tests should be described solely by name; describe more complex techniques in the Methods section.*
☒	☐	A description of all covariates tested
☐	☒	A description of any assumptions or corrections, such as tests of normality and adjustment for multiple comparisons
☐	☒	A full description of the statistical parameters including central tendency (e.g. means) or other basic estimates (e.g. regression coefficient) AND variation (e.g. standard deviation) or associated estimates of uncertainty (e.g. confidence intervals)
☐	☒	For null hypothesis testing, the test statistic (e.g. F, t, r) with confidence intervals, effect sizes, degrees of freedom and P value noted<br>*Give P values as exact values whenever suitable.*
☒	☐	For Bayesian analysis, information on the choice of priors and Markov chain Monte Carlo settings
☒	☐	For hierarchical and complex designs, identification of the appropriate level for tests and full reporting of outcomes
☐	☒	Estimates of effect sizes (e.g. Cohen's *d*, Pearson's *r*), indicating how they were calculated

Our web collection on statistics for biologists contains articles on many of the points above.

Software and code

Policy information about availability of computer code

Data collection	Zeiss ZEN software (black edition) for microscopy, Typhoon FLA 7000 Control software for Southern blot.
Data analysis	Fiji based on ImageJ v1.53 (Schindelin et al, 2012), Cellpose (Stringer et al, 2021), GraphPad Prism v9.5.1, Clustal Omega (Sievers et al, 2011), ESPRIPT (Robert & Gouet, 2014), custom script for image segmentation and DNA foci quantification (https://doi.org/10.5281/zenodo.7740611), custom script for FRAP analysis (https://doi.org/10.5281/zenodo.2654601).

For manuscripts utilizing custom algorithms or software that are central to the research but not yet described in published literature, software must be made available to editors and reviewers. We strongly encourage code deposition in a community repository (e.g. GitHub). See the Nature Portfolio guidelines for submitting code & software for further information.

Data

Policy information about availability of data

All manuscripts must include a data availability statement. This statement should provide the following information, where applicable:

- Accession codes, unique identifiers, or web links for publicly available datasets
- A description of any restrictions on data availability
- For clinical datasets or third party data, please ensure that the statement adheres to our policy

Source data are provided with this paper (https://doi.org/10.5281/zenodo.8430514), including raw and processed microscopy data, uncropped gels and blots images and datasheets containing individual values underlying each plot. Reagents used in this study (plasmids, cell lines) are available upon request.

Research involving human participants, their data, or biological material

Policy information about studies with human participants or human data. See also policy information about sex, gender (identity/presentation), and sexual orientation and race, ethnicity and racism.

Reporting on sex and gender	Not applicable, as this study does not involve human participants.
Reporting on race, ethnicity, or other socially relevant groupings	Not applicable, as this study does not involve human participants.
Population characteristics	Not applicable, as this study does not involve human participants.
Recruitment	Not applicable, as this study does not involve human participants.
Ethics oversight	Not applicable, as this study does not involve human participants.

Note that full information on the approval of the study protocol must also be provided in the manuscript.

Field-specific reporting

Please select the one below that is the best fit for your research. If you are not sure, read the appropriate sections before making your selection.

☒ Life sciences ☐ Behavioural & social sciences ☐ Ecological, evolutionary & environmental sciences

For a reference copy of the document with all sections, see nature.com/documents/nr-reporting-summary-flat.pdf

Life sciences study design

All studies must disclose on these points even when the disclosure is negative.

Sample size	Sample sizes were not pre-determined. For each microscopy session, we acquired the maximum number of images while processing all samples. For each condition, we obtained 15 to 20 fields of view from several independent transfection experiments, which was sufficient to detect statistically significant differences between samples.
Data exclusions	No data were excluded.
Replication	Each experiment was replicated at least two times.
Randomization	Randomization was not possible or relevant, as our experiments involved cultured cell lines.
Blinding	Blinding was not relevant for this study, as there were no prior assumptions about the experimental outcomes. Quantitative measurements on images were automated, and are therefore not affected by prior knowledge of sample identity.

Reporting for specific materials, systems and methods

We require information from authors about some types of materials, experimental systems and methods used in many studies. Here, indicate whether each material, system or method listed is relevant to your study. If you are not sure if a list item applies to your research, read the appropriate section before selecting a response.

Materials & experimental systems

n/a	Involved in the study	
☐	☒	Antibodies
☐	☒	Eukaryotic cell lines
☒	☐	Palaeontology and archaeology
☐	☒	Animals and other organisms
☒	☐	Clinical data
☒	☐	Dual use research of concern
☒	☐	Plants

Methods

n/a	Involved in the study	
☒	☐	ChIP-seq
☒	☐	Flow cytometry
☒	☐	MRI-based neuroimaging

Antibodies

Antibodies used	- Total Histone H3 (Rabbit polyclonal antibody): Abcam cat. ab1791

- H3K9me1 (Mouse monoclonal antibody): Active Motif cat. 39681

- H3K9me2 (Rabbit polyclonal antibody): Jenuwein Laboratory cat. 4677 (see Perez-Burgos et al, 2003)

- H3K9me3 (Rabbit polyclonal antibody): Abcam cat. ab8898

- HP1α (Rabbit polyclonal antibody): Cell Signaling cat. 2616

- MeCP2 (Rabbit monoclonal antibody): Cell Signaling cat. 3456

- TUJ1 (TUBB3) (Mouse monoclonal antibody): BioLegend cat. 801201

- 5-methylcytosine (Mouse monoclonal antibody): Active Motif cat. 39649

Validation

All antibody targets are highly conserved across vertebrates. Therefore, these reagents were expected to react with the various mammalian species used in this study.

- Total Histone H3: This antibody recognises a peptide conserved in multiple variants of Histone H3 such as H3.1, H3.2 and H3.3. For manufacturer's validation, see: https://www.abcam.com/products/primary-antibodies/histone-h3-antibody-nuclear-marker-and-chip-grade-ab1791.html.

- H3K9me1 antibody: For manufacturer's validation, see: https://www.activemotif.com/catalog/details/39681. As expected, no Western-blot signal was detected in cell lines lacking H3K9 methyltransferases (5KO/6KO fibroblasts, see this study and Montavon et al, 2021 (https://doi.org/10.1038/s41467-021-24532-8)).

- H3K9me2 antibody: Information regarding the generation and characterisation of this antibody was reported by Perez-Burgos et al, 2003 (https://doi.org/10.1016/S0076-6879(03)76016-9). As expected, no Western-blot signal was detected in cell lines lacking H3K9 methyltransferases (5KO/6KO fibroblasts, see this study and Montavon et al, 2021 (https://doi.org/10.1038/s41467-021-24532-8)).

- H3K9me3 antibody: For manufacturer's validation, see: https://www.abcam.com/products/primary-antibodies/histone-h3-tri-methyl-k9-antibody-chip-grade-ab8898.html. As expected, no Western-blot or immunofluorescence signal was detected in cell lines lacking H3K9 methyltransferases (2KO/5KO/6KO fibroblasts, see this study and Montavon et al, 2021 (https://doi.org/10.1038/s41467-021-24532-8)).

- HP1α antibody: For manufacturer's validation, see: https://www.cellsignal.com/products/primary-antibodies/hp1a-antibody/2616. As expected, HP1α immunofluorescence signal becomes diffuse in cell lines lacking H3K9 methyltransferases (2KO/5KO fibroblasts, see this study).

- MeCP2 antibody: For manufacturer's validation, see: https://www.cellsignal.com/product/productDetail.jsp?productId=3456. As expected, no immunofluorescence signal was detected in cell lines lacking MeCP2 (Mecp2 knockout fibroblasts, see this study).

- TUJ1 (TUBB3) antibody: TUJ1 recognizes an epitope located within the last 15 C-terminal residues of Class III ß-tubulin (TUBB3). This antibody is highly reactive to neuron-specific TUBB3 and does not cross-react with ß-tubulin found in glial cells. For manufacturer's validation, see: https://www.biolegend.com/en-ie/products/purified-anti-tubulin-beta-3-tubb3-antibody-11580.

- 5-methylcytosine antibody: For manufacturer's validation, see: https://www.activemotif.com/catalog/details/39649. As expected, no immunofluorescence signal was detected in cell lines lacking DNA methyltransferases (DNMT TKO ESCs, see this study).

Eukaryotic cell lines

Policy information about cell lines and Sex and Gender in Research

Cell line source(s)

J1 ESCs (Mus musculus, Sex: Male, Source: (Tsumura et al, 2006))
DNMT TKO ESCs (Mus musculus, Sex: Male, Source: (Tsumura et al, 2006))
Eset25 MEF (Mus musculus, Sex: Unkown, Source: (Montavon et al, 2021))
2KO MEF (Mus musculus, Sex: Unkown, Source: (Montavon et al, 2021))
5KO MEF (Mus musculus, Sex: Unkown, Source: (Montavon et al, 2021))
2-17 MEF (Mus musculus, Sex: Male, Source: (Guy et al, 2001))
2-17X MEF (Mus musculus, Sex: Male, Source: (Guy et al, 2001))
NIH 3T3 (Mus musculus, Sex: Male, Source: ECACC 93061524)
HEK-293 (Human, Sex: Female, Source: ATCC CRL-1573)
LUHMES (Human, Sex: Female, Source: (Shah et al, 2016))
COS-7 (African Green Monkey, Sex: Male, Source: ATCC CRL-1651)
CHO (Chinese Hamster, Sex: Female, Source: ATCC CCL-61)
JH4 clone 1 (Guinea pig, Sex: Female, Source: ATCC CCL-158)
Rat fibroblasts (Sex: Unkown, Source: Dr Tom Burdon (this study))
OEF (Sheep, Sex: Unkown, Source: Prof Bruce Whitelaw (Sartori et al, 2012))
Cow fibroblasts (Sex: Unkown, Source: Dr Tom Burdon (this study))
Pig fibroblasts (Sex: Male, Source: Dr Tom Burdon (this study))
Warthog fibroblasts (Sex: Female, Source: Dr Tom Burdon (this study))
RRH#1 (Red River Hog, Sex: Male, Source: Dr Tom Burdon (this study))
SMG (Cat, Sex: Female, Source: Dr Gura Bergkvist (Gray et al, 2017))
Dog fibroblasts (Sex: Female, Source: Dr Tom Burdon (this study))
RedD-2 (Red Deer, Sex: Female, Source: Dr Tom Burdon (this study))
Roe Deer fibroblasts (Sex: Male, Source: Dr Tom Burdon (this study))

	EEF 1.3 (Horse, Sex: Unkown, Source: Dr Xavier Donadeu (Sharma et al, 2014))<br>Mus spretus fibroblasts (Sex: Unkown, Source: this study)
Authentication	The identity of each cell line was verified by Sanger sequencing of mitochondrial Cytochrome b (Kocher et al, 1989; Irwin et al, 1991). The obtained sequences were compared to reference sequences available on public databases (Ensembl, NCBI) to confirm the exact species attributed to each cell line.
Mycoplasma contamination	All cell lines were tested negative for Mycoplasma contamination.
Commonly misidentified lines<br>(See ICLAC register)	No commonly misidentified lines were used.

Animals and other research organisms

Policy information about studies involving animals; ARRIVE guidelines recommended for reporting animal research, and Sex and Gender in Research

Laboratory animals	The rodents used in this study were bred and maintained at the University of Edinburgh animal facilities under standard conditions with 12h dark/light cycles, an ambient temperature of 20-24°C and relative humidity of 45-65%. All procedures were carried out by staff licensed by the UK Home Office and in accordance with the Animal and Scientific Procedures Act 1986. Brain tissue was harvested from one 4 weeks old Long-Evans hooded wild-type male rat, and from one 10 weeks old wild-type male mouse on a mixed C57BL/6 x CBA background.
Wild animals	No wild animals were used in this study.
Reporting on sex	Both rodents used were male. Collected tissues were used to determine MeCP2 nuclear distribution by immunofluorescence, which is not influenced by the sex animals. Therefore, sex was not considered as a key parameter in the design of our study. We reported the sex of all our animals and cell lines, when this information was known.
Field-collected samples	No field-collected samples were used in this study.
Ethics oversight	All experiments involving mice and rats were approved by the local Animal Welfare Ethical Review Body (AWERB) of the University of Edinburgh and were part of project licenses approved by the UK Home Office (PP4326006 and PP4366223), and in accordance with the Animal and Scientific Procedures Act 1986.

Note that full information on the approval of the study protocol must also be provided in the manuscript.
